# Prevalence and Risk Factors for Post-Discharge Feeding Problems in Children Born Extremely Preterm

**DOI:** 10.1097/MPG.0000000000003704

**Published:** 2023-01-18

**Authors:** Stina Alm, Elisabeth Stoltz Sjöström, Magnus Domellöf

**Affiliations:** From the * Department of Clinical Sciences, Paediatrics, Umeå University, Umeå, Sweden; the † Department of Food, Nutrition and Culinary Science, Umeå University, Umeå, Sweden.

**Keywords:** feeding difficulties, low birth weight, premature infants, stunting, underweight

## Abstract

Preterm infants have a high risk of post-discharge feeding problems, but there is a lack of population-based studies in infants born extremely preterm and little is known about underlying mechanisms. The objectives were to assess the incidence of post-discharge feeding problems and underweight in a population-based cohort of infants born extremely preterm in Sweden (EXPRESS) and identify perinatal risk factors.

**Methods:**

Perinatal health data and prenatal/postnatal growth data was prospectively collected in the cohort. Data on clinical diagnoses related to feeding problems were obtained from the Swedish Patient Register and population prevalence data was also obtained. The main outcome was a composite of post-discharge feeding problem diagnosis and/or underweight at 2.5 years of age.

**Results:**

In total, 66 children (19%) had post-discharge feeding problems diagnosed before 2 years and/or underweight at 2.5 years of age. The risk of feeding problems when compared to the general population was significantly higher, with an odds ratio (OR) of 193 (95% confidence interval (CI) 137.6–270.9). The strongest risk factors for feeding problems were the number of days on mechanical ventilation during the first 8 postnatal weeks, OR of 1.59 (CI 95% 1.29–1.98), and the Clinical Risk Index for Babies-score, OR of 1.14 (CI 95% 1.03–1.26).

**Conclusions:**

Post-discharge feeding problems and underweight are common in children born extremely preterm. The strongest perinatal risk factor for later feeding problems was early treatment with mechanical ventilation. Identifying infants at risk of post-discharge feeding problems might be useful for targeting of nutritional support.

**What Is Known**

- Infants born preterm are at risk of developing feeding problems after discharge.
- Underweight and stunting is commonly observed in very preterm infants at and after discharge.

**What Is New**

- One in 5 infants born extremely preterm was diagnosed with feeding problems after discharge or underweight at 2.5 years of age.
- This study found that the risk of being diagnosed with feeding problems or being underweight at 2.5 years is significantly associated with the number of days treated with mechanical ventilation during the first 8 postnatal weeks.

The last few decades have shown exceptional improvements in the survival rate of infants born extremely preterm (1). However, the burden of morbidities is substantial for these infants and may result in long-term health problems (2). Long-term cognitive, motor, and/or behavioral impairment (3,4) as well as growth failure (5,6) have been described.

Several studies have suggested that preterm infants, especially very preterm infants, are at risk of feeding problems after hospital discharge, including swallowing difficulties, oral motor dysfunction, oral hypersensitivity, eating-behavior disorders, food refusal, difficulty to transition to solid foods, and longer meal durations (4,7–11).

Insufficient nutritional intakes during the first crucial months and years of life affect the growth of the child and, globally, malnutrition is a common cause of stunting (12). In extremely preterm infants, it has been shown that infants who did not show a catch-up growth by the age of 2 years have an increased risk of remaining below both weight and height averages as they reach early youth (5).

Various hypotheses have been proposed on the causal mechanisms resulting in later feeding problems. Prolonged treatment with feeding tubes and endotracheal intubation has been shown to be associated with impaired oral motor development, altered oral sensitivity, altered palatal development (palatal groove), and delayed feeding development in premature infants (10,13,14). An effect on appetite regulation is also possible, even though this has been less studied (15).

It has been described that preterm infants have a high risk of feeding problems after discharge, but there is a lack of population-based studies in infants born extremely preterm and little is known on clinical risk factors and underlying mechanisms. The objectives of this study were to assess the incidence of feeding problems up to 2 years of age in a population-based cohort of infants born before 27 gestational weeks in Sweden (EXPRESS) and to identify perinatal risk factors for later feeding problems and underweight at 2.5 years of age.

## METHODS

### Study Population

**FIGURE 1.**
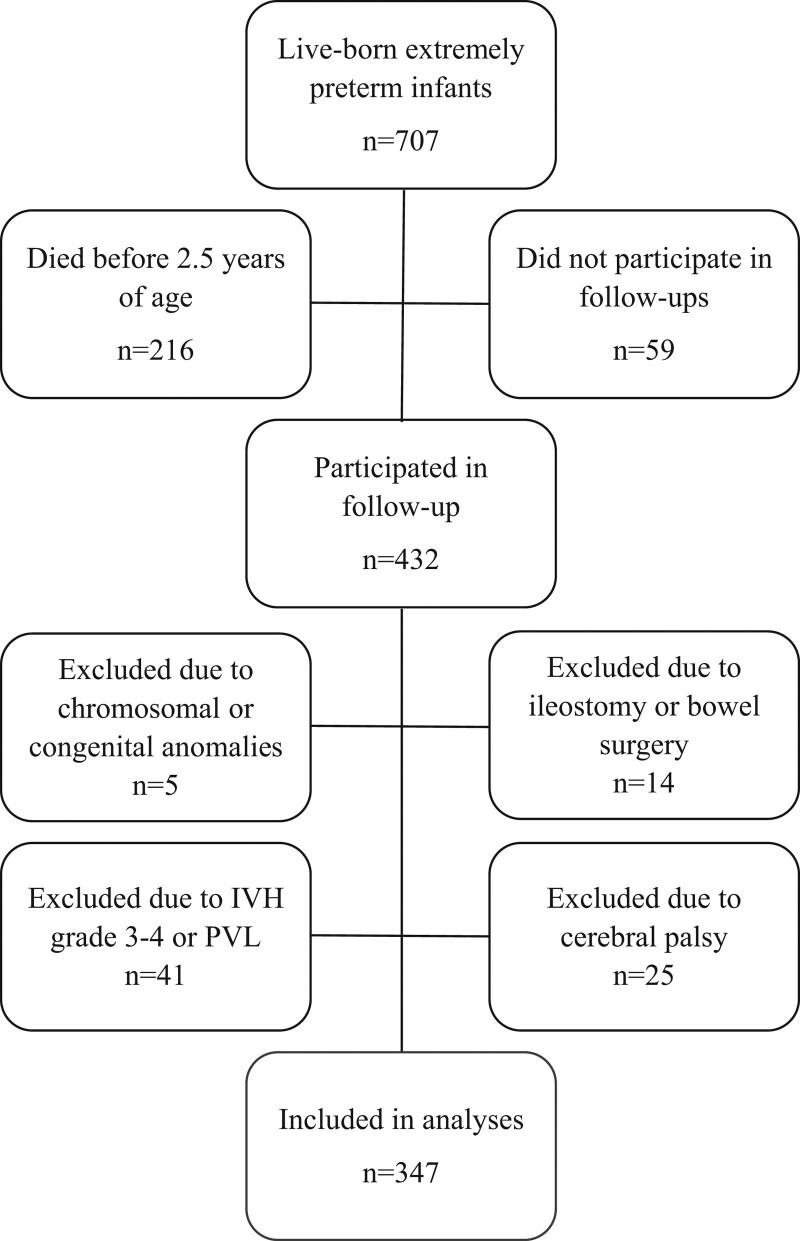
Flowchart of study population. IVH = intraventricular hemorrhage; PVL = periventricular leucomalacia.

The study population originate from the EXPRESS cohort, which included all infants born before 27 completed gestational weeks between April 1, 2004 and March 31, 2007 (16). Of 707 live born, 432 children took part in the follow-up at 2.5 and 6.5 years of age (17). For infants not included in this study a majority (n = 216) died before 2.5 years of age, whereas a minority (n = 59) did not participate in follow ups (Fig. 1).

Infants with major congenital or chromosomal anomalies known to affect weight and height were excluded (n = 5), as well as infants with ileostomy or that underwent bowel surgery (n = 14). The most common chromosomal anomaly was Trisomy 21. The reason for infants to undergo bowel surgery or had ileostomies was due to diagnosed or suspected necrotizing enterocolitis (NEC). We also excluded infants with neurological disorders that might affect feeding problems because of the effect on the developing brain: those diagnosed with intraventricular hemorrhage stage 3 and 4 and/or periventricular leucomalacia (n = 41), as well as infants who were diagnosed with cerebral palsy according to stage 2 or higher in the gross motor function classification system (GMFCS, n = 25). GMFCS is a validated system used to differentiate a child with cerebral palsy’s abilities, limitations, and need for assistive technologies (18,19). In total, 347 infants were included in analyses (Fig. 1).

### Defining Feeding Problems

In this study, feeding problems were defined as having 1 or more of a number of diagnoses from the International Statistical Classification of Diseases and Related Health Problems – Tenth Revision (ICD-10). In order to cover all relevant ICD-10 codes, we included codes related to feeding behavior (R63.3 and F98.2), failure to thrive and malnutrition (R62 and E43), dysphagia (R13.9), and need of nutritional support (nasogastric tube >6 months and percutaneous endoscopic gastrostomy) (Table 1, Supplemental Digital Content 1, http://links.lww.com/MPG/D51). Additionally, we wanted to examine the extent of interventions, and therefore included possible interventions related to feeding problems, also presented in Table 1, Supplemental Digital Content 1, http://links.lww.com/MPG/D51. Since all children with feeding problems may not have received a diagnosis, we decided to also include those with underweight [below −2 standard deviation (SD)] at 2.5 years in a composite outcome variable “feeding problems and/or underweight,” henceforth abbreviated as FPUW.

### Data Collection

Feeding problem-related diagnoses between discharge and 2 years of age were collected from the National Patient Register (Swedish National Board of Health and Welfare). Aggregated diagnosed data from the whole Swedish population during the same period were also obtained from the National Patient Register.

Growth data were collected at birth, 36 weeks postmenstrual age (PMA) and at 2.5 years of age. Data was retrieved from hospital records and from the EXPRESS cohort database. A Swedish gender-specific growth reference was used to calculate the standard deviation scores (SDS) for weight and length (20). For data on weight and length at 36 weeks PMA we accepted measurements taken within a period of ±2 days for weight and ±4 days for length. The WHO growth reference (21) was used for growth data at 2.5 years of age.

Small for gestational age (SGA) was defined as a birth weight more than 2 SD below mean. Underweight was defined as weight-for-age <−2 SD. Stunting was defined as length/height-for-age <−2 SD. A weight and length/height between −2 SD and +2 SD were considered to be appropriate for age (AGA).

Data on perinatal and morbidity data included Apgar-scores, Clinical Risk Index for Babies (CRIB)-score, antenatal steroids, respiratory support, postnatal steroids, and treatment with antibiotics. Exact time intervals for neonatal treatments were collected prospectively.

### Statistical Analysis

The primary composite outcome, FPUW, was a combination of post-discharge diagnoses/interventions (up to 2 years of age) and underweight at 2.5 years. FPUW was defined as having at least 1 of the diagnoses or interventions listed in Table 1, Supplemental Digital Content 1, http://links.lww.com/MPG/D51 after discharge and before the age of 2 years uncorrected age or having a weight below −2 SD at 2.5 years.

We included the following variables as potential risk factors for FPUW: birth weight, birth weight SDS, CRIB-score, Apgar score at 10 minutes, days on mechanical ventilation, severe bronchopulmonary dysplasia (BPD), treatment with postnatal steroids, and treatment with antibiotics.

First, we analyzed the associations between these risk factors and FPUW in univariable binary logistic regressions. Finally, the significant variables from the univariable analyses were entered into a binary logistic regression model using a stepwise approach. A receiver operating characteristic (ROC) analysis was performed to assess the number of days with mechanical ventilation as a predictor for the risk of later feeding problems. As a final step we redid the multivariable analysis using only the diagnosed feeding problems as the outcome.

The statistical analyses were performed by using SPSS Statistical software version 25.0 (IBM Corp, New York, NY). A *P* value of less than 0.05 was considered statistically significant. The Regional Ethical Review Board in Umeå, Sweden, approved the study (Dnr 2017/425-31).

## RESULTS

Of the 432 infants that took part in the follow up program, 347 were included in the analyses (Fig. 1). Of the included infants, 14.1% (n = 49) were born SGA and 47.8% (n = 166) were girls. The mean gestational age at birth was 25.5 weeks. Anthropometric characteristics of the study population are presented in Table 2, Supplemental Digital Content 2, http://links.lww.com/MPG/D52. All infants had a recorded birth weight, whereas birth length was available for 80% of the infants. The mean duration of mechanical ventilation was 11.97 days, mean CRIB-score 5.7, and 25% of the included infants received postnatal steroids (for a mean 3.48 days). Severe BPD was diagnosed in 28% of the infants. Apgar-scores at 1, 5, and 10 minutes in mean were 5.53, 7.44, and 8.46, respectively.

### Incidence of Feeding Problems

In this cohort, 66 infants (19%) had the composite outcome FPUW between discharge and 2 years and/or underweight at 2.5 years (Table 1). Of these, 7 infants were underweight at 2.5 years but had not been given any of the listed diagnoses in Table 1, Supplemental Digital Content 1, http://links.lww.com/MPG/D51 regarding feeding problems. Furthermore, 48 of the 66 infants (81%) had been given at least 1 of the diagnoses after discharge but were not underweight at 2.5 years of age. Of all infants, 19 (28.8%) had been given at least 2 of the listed diagnoses in Table 1, Supplemental Digital Content 1, http://links.lww.com/MPG/D51. The most common diagnosis was R62 which includes failure to thrive and stunting with 5.8% (n = 20) of the infants in the cohort. The most frequent intervention was the use of nasogastric tube with a duration of more than 6 months (6.6%, n = 23). Table 1 presents detailed data for each of the included diagnosis and interventions.

**TABLE 1.** Incidence of feeding problems in children born extremely preterm after discharge until 2 years and underweight at 2.5 years of age

	n (%)	Mean age at diagnosis (mo)	Mean GA	Mean birth weight (*z*-birth weight)	Mean birth length (*z*-birth length)	ΔSDS-weight until 36 weeks GA (mean)	ΔSDS-length until 36 weeks GA (mean)	Mean weight (kg) at 2.5 years (*z*-weight)	Mean length at 2.5 years (*z*-length)	Days of MV during first 8 weeks of life (mean)
Diagnosis										
Unspecified severe protein-energy malnutrition (E43)	3 (0.9)	11.3	24.40	549 (−1.87)	30.0 (−2.34)	−2.20	−3.97	11.1 (−1.38)	85.5 (−1.63)	34
Feeding disorder of infancy and childhood (F98.2)	5 (1.2)	12.8	25.14	612 (−2.11)	30.5 (−2.30)	−1.65	−3.47	11.0 (−1.57)	86.1 (−1.55)	29
Feeding problems of new-born (P92)	10 (2.9)	6.6	25.84	786 (−1.19)	31.7 (−2.62)	−1.61	−2.69	11.9 (−0.83)	87.5 (−1.04)	17
Other lack of expected normal physiological development (R62)	20 (5.8)	13.7	24.95	677 (−1.29)	30.7 (−1.89)	−1.79	−3.67	11.0 (−1.49)	86.7 (−1.34)	25
Feeding difficulties and mismanagement (R63)	18 (5.2)	12.2	25.34	776 (−0.78)	32.5 (−1.34)	−1.51	−3.33	11.6 (−1.03)	88.8 (−0.75)	21
Dysphagia (R13.9)	4 (1.2)	20	25.65	755 (−1.09)	32.3 (−2.17)	−1.06	−4.13	11.3 (−0.96)	84.3 (−1.80)	22
Underweight at 2.5 years (weight <−2 SD)	18 (5.2)	N/A	25.12	627 (−1.95)	30.4 (−2.63)	−1.17	−2.32	9.56 (−2.67)	83.64 (−2.28)	26
Intervention										
PEG	12 (3.5)	14.8	25.0	650 (−1.61)	30.5 (−2.50)	−1.75	−3.36	12.1 (−0.64)	86.3 (−1.41)	32
Nasogastric tube >6 mo	23 (6.6)	N/A	24.99	696 (−1.09)	31.7 (−1.71)	−1.34	−2.35	11.9 (−0.88)	87.7 (−1.07)	21
Any of the above diagnoses or underweight (FPUW)	66 (19.0)	N/A	25.06	704 (−1.10)	31.4 (−1.81)	−1.48	−2.78	11.6 (−1.08)	87.5 (−1.12)	22
None of the above diagnoses or interventions	281 (81.0)	N/A	25.61	807 (−0.70)	33.3 (−1.12)	−1.29	−2.06	13.1 (−0.04)	90.3 (−0.29)	10

FPUW = feeding problems after discharge until 2 years and/or underweight at 2.5 years composite outcome variable; GA = gestational age; MV = mechanical ventilation; PEG = percutaneous endoscopic gastrostomy; SDS = standard deviation scores.

The risk of feeding problems diagnoses/interventions among children born extremely preterm was significantly higher than in the general pediatric population (17% vs 0.11%), with an odds ratio (OR) of 193.1 (95% CI 137.6–270.9).

### Univariable Associations Between Neonatal Risk Factors and FPUW

Associations between neonatal risk factors and FPUW were first investigated using univariable linear analyses (Table 2). The number of days treated with mechanical ventilation was significantly associated with the risk of later feeding problems/underweight for each week tested (postnatal week 1–8), as well as for the entire period of the first 8 postnatal weeks (*P* ≤ 0.001). Being treated with postnatal steroids was also significantly associated with the risk of FPUW for all the first 8 postnatal weeks, except week 1, and was also significant when considering the whole period of 8 weeks (*P* ≤ 0.001). Treatment with antibiotics was not significantly associated with later outcome for any of the first 8 postnatal weeks. Severe BPD was significantly associated with the composite outcome FPUW (*P* ≤ 0.001), as well as lower gestational age at birth (*P* ≤ 0.001). A low Apgar score at 10 minutes and a high CRIB-score was significantly associated with FPUW (*P* = 0.021 and *P* ≤ 0.001, respectively).

**TABLE 2.** Infant characteristics and univariable binary logistic regressions for risk factors

	Mean (SD) or %	Range	OR	*P* value
Gestational week at birth	25.5 (1.06)	22.1–26.9	0.63	<0.001
Apgar score at 10 min	8.46 (1.49)	3–10	0.82	0.021
CRIB-score	5.7 (3.33)	1–16	1.24	<0.001
Antenatal steroids*	89.90%		0.9	0.781
Severe bronchopulmonary dysplasia (>30% oxygen at 36 weeks PMA)	28%		2.6	<0.001
Treated with mechanical ventilation†	82.70%			
Days treated with mechanical ventilation†,‡	11.97 (13.27)	0–56	1.06	<0.001
Treatment with postnatal steroids†	25.10%			
Days treated with postnatal steroids†,‡	3.48 (8.13)	0–51	1.06	<0.001
Treatment with antibiotics†	74.90%			
Days with antibiotic treatment†,‡	8.28 (6.40)	0–30	0.99	0.709

CRIB = Clinical Risk Index for Babies; OR = odds ratio; PMA = postmenstrual Age; SD = standard deviation.

* One or two doses.

† In the first 8 postnatal weeks.

‡ In the treated group.

### Multivariable Associations Between Neonatal Risk Factors and FPUW

In the multivariable analysis, variables that were significant in univariable analysis were included. Two significant variables remained in the multivariable analysis; the number of days treated with mechanical ventilation between birth and 8 weeks of age (OR = 1.048, *P* ≤ 0.001) and CRIB-score (OR = 1.142, *P* = 0.009). The risk of having FPUW increased by 4.8% for each day treated with mechanical ventilation during the first 8 postnatal weeks. The significant association between days on mechanical ventilation and later feeding problems remained when using only diagnosed feeding problems as the outcome (OR 1.057, *P* ≤ 0.001).

The ROC analysis showed that the number of days on mechanical ventilation was a significant predictor of FPUW with an area under the curve of 0.73 (95% CI 0.652–0.797). The cut-off with the highest combination of sensitivity and specificity was 10 days on mechanical ventilation, which had a sensitivity of 71% (0.712) and a specificity of 67% (0.665).

No significant interaction between FPUW and the method of intubation during the first 2 postnatal weeks (*P* = 0.140) were found.

When children with diagnosed feeding problems were removed from the multivariable analysis, thus only examining the children with underweight at 2.5 years, a significant association between days on mechanical ventilation and either underweight (OR 1.059, *P* ≤ 0.001) or stunting (OR 1.045, *P* = 0.001) at 2.5 years of age remained.

### Growth Analyses

Anthropometric data was available for all 3 occasions studied (birth, 36 weeks PMA, and at 2.5 years of age) in 278 (80%) infants regarding weight, and 180 (52%) infants regarding length/height.

**FIGURE 2.**
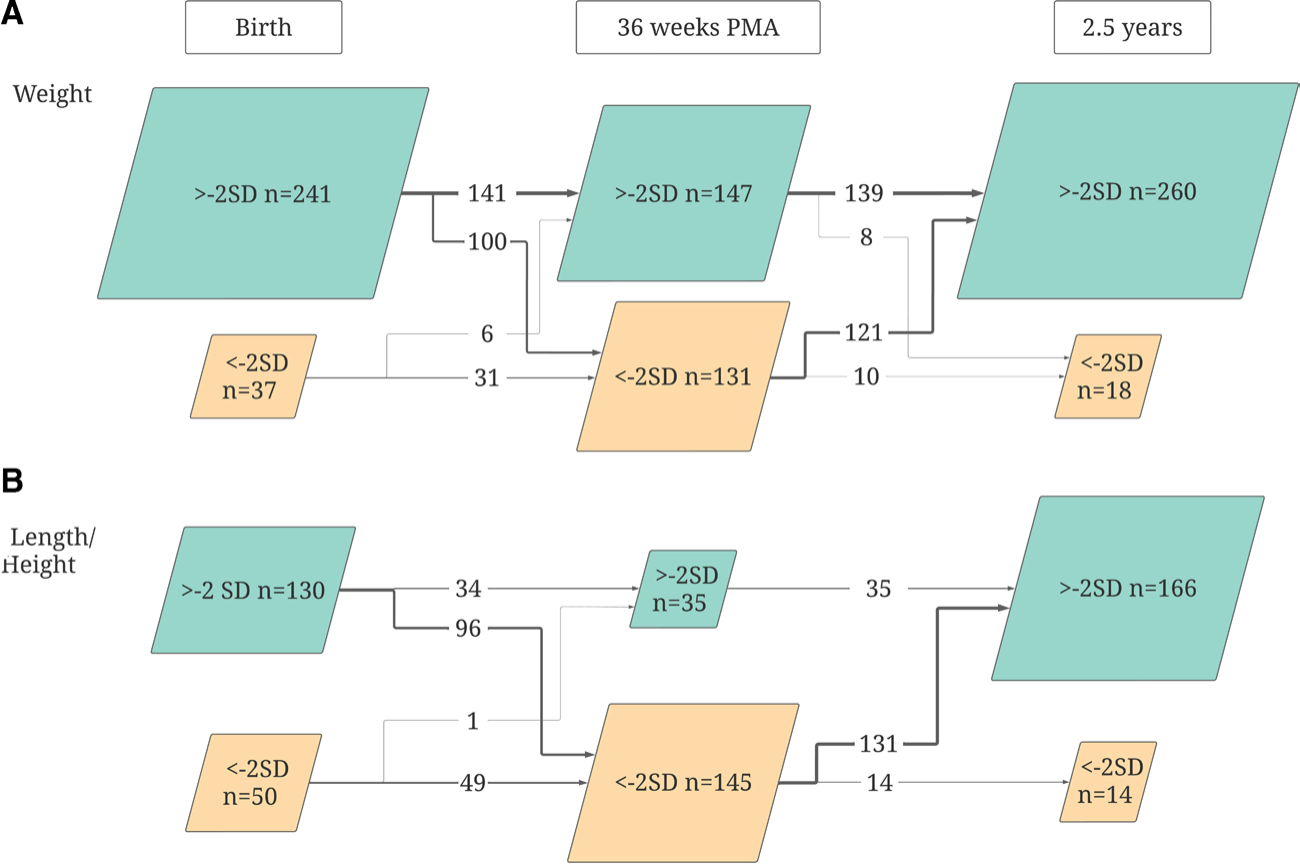
Weight (A) and length/height (B) change in standard deviations from birth to 2.5 years of age in children born extremely preterm (A; n = 278, B; n = 180). PMA = postmenstrual age; SD = standard deviation.

The proportions of infants with normal weight (–2 SD to +2 SD) and underweight at the different ages are illustrated in Figure 2A and a similar flowchart for length/height is shown in Figure 2B. The arrows describe numbers of infants that are changing different SDS at each time period.

A majority (87%) of the infants had a weight appropriate for gestational age at birth. However, at the age 36 weeks PMA, 42% of the infants born AGA were underweight (below −2 SD). Almost all of these infants showed a progress in weight gain at 2.5 years where 93% of the children had a weight above −2 SD.

The same pattern, but even more pronounced, was present when observing the change in SDS regarding length/height, 72% of the infants had a normal birth length (above −2 SD), but at 36 weeks PMA, 81% were stunted (below −2 SD). At 2.5 years of age, 92% of the children had a normal height (above −2 SD).

## DISCUSSION

### Feeding Problems

In this cohort of extremely preterm infants, the incidence of feeding problems after discharge from neonatal intensive care units (NICUs) was 19%, using our composite outcome, FPUW, including feeding problem diagnosis and/or underweight up to 2.5 years of age. This incidence is similar compared with other studies on premature infants using various definitions of feeding problems, which have found incidences ranging between 20% and 38% (7,22,23). Furthermore, we found a significantly increased risk of feeding problems diagnoses in children born extremely preterm when compared to the general population (OR 193.1, 95% CI 137.6–270.9).

Sanchez et al (7) examined a population of preterm infants born before 30 completed gestational weeks compared with term-born controls at 12 months’ corrected age, using the Schedule for Oral Motor Assessment. They found that the preterm infants had an increased risk of having oromotor feeding difficulties, including food textures and swallowing, with an OR of 2.21 (95% CI 1.55–3.16), and 38% of the premature infants had feeding problems(7).

A Dutch study by Hoogewerf et al (23) compared the prevalence of feeding problems at 1–2 years of age in infants treated at level III/IV NICU for at least 4 days, and found that all infants treated regardless of gestational age had a higher prevalence of feeding problems (20.4%) than a group of term-born controls. Surprisingly, they found no difference in the prevalence of feeding problems among infants with different gestational ages at birth within the group treated at the NICU. Feeding data was reported by the parents using a questionnaire, which was a translated version of the Montreal Children’s Hospital Feeding Scale developed by Ramsay et al (24).

The incidence of post-discharge feeding problems in our study is likely an underestimation since it is based on register data and a single anthropometric measurement at 2.5 years of age. Thus, the true incidence in our cohort is likely to be at least 19%.

Our study shows that a large proportion of extremely preterm infants have postnatal growth impairments leading to underweight during the hospital stay, results similar with Horbar et al (25). At 2.5 years of age most children in our current study had a weight (94%) and height (92%) above −2 SD, even though the proportion of children who were underweight and stunted was almost 3 times the expected in a normal population. Thus, we can see that the infants born before 27 weeks of gestation have a catch-up growth after discharge. This finding is expected given that earlier studies have shown that preterm infants have a catch-up growth during the first years of life. Toftlund et al (26) demonstrated that 56% of very preterm (<32 weeks) had a catch-up growth, defined as an increase by 1 SD, up to 2 years of age. However, as shown by Wiechers et al (27) in another cohort of children born <28 weeks or <1000 g the SDS for weight and body mass index still remained lower than the reference population at 5 years of age. This is consistent with the findings in this study.

In the univariable analysis we found associations between CRIB-score, postnatal steroid treatment, severe BPD, and low Apgar at 10 minutes. A common factor for these is that they all in some way reflect general neonatal morbidity and respiratory challenges in particular. CRIB-score is calculated based on gestational week at birth and birth weight as well as oxygen treatment and maximum base excess during the first 12 hours and the presence of congenital malformations. The total CRIB-score is therefore strongly influenced by the gestational age at birth and reflects general morbidity associated with extremely preterm birth. We found that the number of days treated with mechanical ventilation was the strongest predictor of later feeding problems after discharge, and the risk increased by 4.8% for each day of treatment. The risk factor of being treated more than 10 days on mechanical ventilation had a sensitivity of 71% and a specificity of 73% of predicting later feeding problems. Our results agree with Poore et al (10), who found that preterm infants who required endotracheal intubation and more than 1 week of oxygen treatment showed negatively affected suck patterns by increasing the proportion of nonnutritive sucks. Worth to note is that the mean duration on mechanical ventilation in that study was 6.4 days, which was substantially lower than the mean of 12.0 days in our cohort, reflecting the difference in mean gestational age at birth between studies. In the study by Poore et al (10), the mean birth gestational age was 30.1 weeks as compared to 25.5 weeks in our study. It was expected to find that neonatal morbidity was associated with the risk of later feeding problems, but it was unexpected that the days treated with mechanical ventilation was such a strong risk factor. Possible underlying mechanisms include effect on oral-motor development caused by the endotracheal tube, or it could reflect a more general effect of severe respiratory disease.

Enomoto et al reported a correlation between oral endotracheal intubation and the development of acquired palatal grooves in extremely and very low birth weight infants in Japan (13). Their result was significant after adjustment for gestational age, birth weight, and the number of days of oral duodenal tube placement. The development of a palatal groove was shown to be associated with delayed establishment of oral feeding. In our cohort, the majority of infants were nasally intubated (77%) and we found no significant interaction between the method of intubation (nasal vs oral) and later feeding problems.

Prolonged use of feeding tubes is crucial for supplying nutrition to extremely preterm infants. However, it has been suggested that the presence of feeding tubes might hinder oral feeding attempts and affect the suck-swallow-breath coordination (28). One study has shown that tube feeding is also linked to increased gastroesophageal reflux in preterm infants (29). In our cohort, we found that 23 infants (6.6%) still had a feeding tube at 6 months of age. Unfortunately, we lack data on the use of feeding tubes in the NICU during the studied years, so we were unable to analyze the possible effects on later feeding problems.

Our finding of a significant and strong correlation between days with treatment of mechanical ventilation and the post-discharge feeding problems is of potentially great significance for the development of post-discharge follow-up programs of extremely preterm infants. Infants who are treated with mechanical ventilation for a longer period than 10 days might benefit from planned early visits with speech therapists and dietitians to identify and treat feeding problems early. This could also help prevent later stunting or underweight in this vulnerable patient group that is already at risk for poor growth (5).

### Strengths and Limitations

The strengths of this study were the prospective inclusion of all infants born before 27 gestational weeks in Sweden. Also, the data on diagnoses were collected from the Swedish National Board of Health and Welfare from the National Patient Register which undergoes ongoing quality controls and has been shown to be of high data quality (5). However, the National Patient Register is only as accurate as the data reported to it, and we have attempted to minimize the risk of missing infants with relevant diagnoses by including various diagnoses related to feeding problems. The selection of diagnoses was done together with active clinicians, dietitians, and speech therapists to cover the various professions reporting diagnoses to the register. Although the children in this cohort were born during the first decade of this millennium, we believe that the fundamental risk factors for later feeding problems are the same as today. Even though the prevalence of feeding problems and underweight may have changed over time the current study is valuable as a comparison when evaluating contemporary clinical practices.

## CONCLUSIONS

This study shows that the risk of being diagnosed with feeding problems, or being underweight at 2.5 years, is significantly associated with the number of days treated with mechanical ventilation during the first 8 postnatal weeks. We propose that all infants who are discharged from NICUs and have spent more than 10 days intubated could potentially benefit from a follow-up program involving for example physicians, dietitians, and speech therapists to intervene early and help these children avoid later growth restrictions and morbidities.

## Supplementary Material



## References

[1] Field DJ Dorling JS Manktelow BN Draper ES. Survival of extremely premature babies in a geographically defined population: prospective cohort study of 1994-9 compared with 2000-5. BMJ 2008;336:1221–3. 18469017 10.1136/bmj.39555.670718.BE PMC2405852

[2] Norman M Hallberg B Abrahamsson T. Association between year of birth and 1-year survival among extremely preterm infants in Sweden during 2004-2007 and 2014-2016. JAMA 2019;321:1188–99. 30912837 10.1001/jama.2019.2021 PMC6439685

[3] Serenius F Kallen K Blennow M. Neurodevelopmental outcome in extremely preterm infants at 2.5 years after active perinatal care in Sweden. JAMA 2013;309:1810–20. 23632725 10.1001/jama.2013.3786

[4] Samara M Johnson S Lamberts K Marlow N Wolke D. Eating problems at age 6 years in a whole population sample of extremely preterm children. Dev Med Child Neurol 2010;52:e16–22. 19832883 10.1111/j.1469-8749.2009.03512.x

[5] Farooqi A Hagglof B Sedin G Gothefors L Serenius F. Growth in 10- to 12-year-old children born at 23 to 25 weeks’ gestation in the 1990s: a Swedish national prospective follow-up study. Pediatrics 2006;118:e1452–65. 17079546 10.1542/peds.2006-1069

[6] Stoltz Sjostrom E Ohlund I Ahlsson F. Nutrient intakes independently affect growth in extremely preterm infants: results from a population-based study. Acta Paediatr 2013;102:1067–74. 23855971 10.1111/apa.12359

[7] Sanchez K Spittle AJ Slattery JM Morgan AT. Oromotor feeding in children born before 30 weeks’ gestation and term-born peers at 12 months’ corrected age. J Pediatr 2016;178:113–118.e1. 27609073 10.1016/j.jpeds.2016.07.044

[8] Migraine A Nicklaus S Parnet P. Effect of preterm birth and birth weight on eating behavior at 2 y of age. Am J Clin Nutr 2013;97:1270–7. 23615831 10.3945/ajcn.112.051151

[9] Dodrill P McMahon S Ward E Weir K Donovan T Riddle B. Long-term oral sensitivity and feeding skills of low-risk pre-term infants. Early Hum Dev 2004;76:23–37. 14729160 10.1016/j.earlhumdev.2003.10.001

[10] Poore M Barlow SM Wang J Estep M Lee J. Respiratory treatment history predicts suck pattern stability in preterm infants. J Neonatal Nurs 2008;14:185–92. 19956344 10.1016/j.jnn.2008.07.006 PMC2614286

[11] Pineda R Prince D Reynolds J Grabill M Smith J. Preterm infant feeding performance at term equivalent age differs from that of full-term infants. J Perinatol 2020;40:646–54. 32066844 10.1038/s41372-020-0616-2 PMC7117861

[12] Victora CG de Onis M Hallal PC Blossner M Shrimpton R. Worldwide timing of growth faltering: revisiting implications for interventions. Pediatrics 2010;125:e473–80. 20156903 10.1542/peds.2009-1519

[13] Dodrill P. Feeding difficulties in preterm infants. Infant Child Adolesc Nutr 2011;3:324–31.

[14] Enomoto M Sezaki H Muranishi R. Acquired palatal groove and delayed oral feeding in preterm infants. Pediatr Int 2017;59:171–5. 27501257 10.1111/ped.13113

[15] Savino F Lupica MM Liguori SA Fissore MF Silvestro L. Ghrelin and feeding behaviour in preterm infants. Early Hum Dev 2012;88:Suppl 1:S51-5. 10.1016/j.earlhumdev.2011.12.028 22285781

[16] Fellman V Hellstrom-Westas L Norman M. One-year survival of extremely preterm infants after active perinatal care in Sweden. JAMA 2009;301:2225–33. 19491184 10.1001/jama.2009.771

[17] Serenius F Ewald U Farooqi A. Neurodevelopmental outcomes among extremely preterm infants 6.5 years after active perinatal care in Sweden. JAMA Pediatr 2016;170:954–63. 27479919 10.1001/jamapediatrics.2016.1210

[18] Palisano R Rosenbaum P Walter S Russell D Wood E Galuppi B. Development and reliability of a system to classify gross motor function in children with cerebral palsy. Dev Med Child Neurol 1997;39:214–23. 9183258 10.1111/j.1469-8749.1997.tb07414.x

[19] Palisano RJ Rosenbaum P Bartlett D Livingston MH. Content validity of the expanded and revised gross motor function classification system. Dev Med Child Neurol 2008;50:744–50. 18834387 10.1111/j.1469-8749.2008.03089.x

[20] Niklasson A Albertsson-Wikland K. Continuous growth reference from 24th week of gestation to 24 months by gender. BMC Pediatr 2008;8:8. 18307822 10.1186/1471-2431-8-8 PMC2294116

[21] WHO Multicentre Growth Reference Study Group. WHO Child Growth Standards based on length/height, weight and age. Acta Paediatr Suppl 2006;450:76–85. 16817681 10.1111/j.1651-2227.2006.tb02378.x

[22] Crapnell TL Rogers CE Neil JJ Inder TE Woodward LJ Pineda RG. Factors associated with feeding difficulties in the very preterm infant. Acta Paediatr 2013;102:e539–45. 23952198 10.1111/apa.12393 PMC3873367

[23] Hoogewerf M Ter Horst HJ Groen H Nieuwenhuis T Bos AF van Dijk MWG. The prevalence of feeding problems in children formerly treated in a neonatal intensive care unit. J Perinatol 2017;37:578–84. 28102855 10.1038/jp.2016.256

[24] Ramsay M Martel C Porporino M Zygmuntowicz C. The Montreal Children’s Hospital Feeding Scale: a brief bilingual screening tool for identifying feeding problems. Paediatr Child Health 2011;16:147–e17. 22379377 10.1093/pch/16.3.147 PMC3077303

[25] Horbar JD Ehrenkranz RA Badger GJ. Weight growth velocity and postnatal growth failure in infants 501 to 1500 grams: 2000-2013. Pediatrics 2015;136:e84–92. 26101360 10.1542/peds.2015-0129

[26] Toftlund LH Halken S Agertoft L Zachariassen G. Catch-up growth, rapid weight growth, and continuous growth from birth to 6 years of age in very-preterm-born children. Neonatology 2018;114:285–93. 30011395 10.1159/000489675

[27] Wiechers C Doll JN Maas C. Enteral feeding advancement and growth until 5 years in extremely preterm infants. BMC Pediatr 2021;21:1–420. 34556084 10.1186/s12887-021-02878-8 PMC8459503

[28] Shiao SY Youngblut JM Anderson GC. Nasogastric tube placement: effects on breathing and sucking in very-low-birth-weight infants. Nurs Res 1995;44:82–8. 7892144

[29] Peter CS Wiechers C Bohnhorst B. Influence of nasogastric tubes on gastroesophageal reflux in preterm infants: a multiple intraluminal impedance study. J Pediatr 2002;141:277–9. 12183728 10.1067/mpd.2002.126298

