# Supplementary material for: Prevalence and Risk Factors for Post-Discharge Feeding Problems in Children Born Extremely Preterm
Source: J Pediatr Gastroenterol Nutr. 2023 Jan 18;76(4):498–504. doi: 10.1097/MPG.0000000000003704 (PMC10013140; doi:10.1097/MPG.0000000000003704)
Supplement: Supplementary file 1 [file mpg-76-498-s001.pdf]

Table, Supplemental Digital Content 1 Included diagnoses and interventions, with corresponding ICD-10 code in children born extremely preterm.

Diagnoses	Unspecified severe protein-energy malnutrition (E43)
	Feeding disorder of infancy and childhood (F98.2)
	Feeding problems of new-born (P92)
	Other lack of expected normal physiological development (R62)
	Feeding difficulties and mismanagement (R63.3)
	Dysphagia (R13.9)
Interventions	Percutaneous Endoscopic Gastrostomy (PEG)
	Nasogastric tube > 6 months

ICD-10; International Statistical Classification of Diseases and Related Health Problems - Tenth Revision.
