# Supplementary material for: Prevalence and Risk Factors for Post-Discharge Feeding Problems in Children Born Extremely Preterm
Source: J Pediatr Gastroenterol Nutr. 2023 Jan 18;76(4):498–504. doi: 10.1097/MPG.0000000000003704 (PMC10013140; doi:10.1097/MPG.0000000000003704)
Supplement: Supplementary file 2 [file mpg-76-498-s002.pdf]

Table, Supplemental Digital Content 2. Anthropometric characteristics at birth, 36 weeks postmenstrual age and at 2.5 years of age.

	At birth			36 weeks PMA			2.5 years		
	**n**	**Mean (SD)**	**Range**	**n**	**Mean (SD)**	**Range**	**n**	**Mean (SD)**	**Range**
GA, weeks	347	25.5 (1.06)	22.1 to 26.9						
Weight									
Grams	347	787 (170.5)	361 to 1315	332	2161 (352.0)	970 to 2980	293	12.76 (1.8)	7.9 to 18.5
SDS	347	-0.77 (1.2)	-6.4 to 1.7	332	-2.11 (1.4)	-8.5 to 0.5	293	-0.26 (1.1)	-4.3 to 2.8
Length/Height									
cm	277	32.97 (2.5)	25.7 to 40.0	260	42.5 (2.6)	31.6 to 49.0	290	89.75 (4.0)	77.6 to 103.5
SDS	277	-1.25 (1.6)	-8.1 to 2.6	260	-3.47 (1.7)	-10.8 to 0.8	290	-0.46 (1.2)	-4.2 to 3.4

PMA; postmenstrual age, GA; gestational age, SDS; standard deviation scores
